# Vitamin Supplementation in Sports: A Decade of Evidence-Based Insights

**DOI:** 10.3390/nu18020213

**Published:** 2026-01-09

**Authors:** Magdalena Wiacek, Emilia Nowak, Piotr Lipka, Remigiusz Denda, Igor Z. Zubrzycki

**Affiliations:** Department of Medical and Health Sciences, Radom University, Chrobrego 27, 26-600 Radom, Poland; 117442@student.uthrad.pl (E.N.); 117472@student.uthrad.pl (P.L.); 117356@student.uthrad.pl (R.D.); i.zubrzycki@urad.edu.pl (I.Z.Z.)

**Keywords:** vitamins, sports performance, supplementation, recovery, injury prevention

## Abstract

**Background**: Vitamins are micronutrients involved in multiple physiological processes critical for athletic performance. Because athletes are often exposed to increased oxidative stress, higher metabolic turnover, and greater nutritional demands, which can potentially lead to deficiencies in vitamins, understanding vitamin supplementation as a function of sport discipline is of fundamental importance. **Methods**: This narrative review synthesizes research findings from the past decade, supplemented with earlier studies where necessary, focusing on vitamins A, C, D, E, and the B-complex vitamins. Peer-reviewed literature was evaluated for evidence on the prevalence of deficiencies in athletes, physiological mechanisms, supplementation strategies, and their effects on performance, injury prevention, and recovery. **Results**: Vitamin D deficiency is highly prevalent among athletes, particularly in indoor sports and during the winter months. Supplementation has been shown to improve musculoskeletal health and potentially reduce injury risk. The antioxidant vitamins C and E can attenuate exercise-induced oxidative stress and muscle damage; however, excessive intake may impair adaptive responses such as mitochondrial biogenesis and protein synthesis. Vitamin A contributes to immune modulation, metabolic regulation, and mitochondrial function, while B-complex vitamins support energy metabolism and red blood cell synthesis. **Conclusions**: Vitamin supplementation in athletes should be individualized, targeting confirmed deficiencies and tailored to sport-specific demands, age, sex, and training intensity. Dietary optimization should remain the primary strategy, with supplementation serving as an adjunct when intake is insufficient. Further high-quality, sport-specific, and long-term studies are needed to establish clear dosing guidelines and to assess the balance between performance benefits and potential risks associated with over-supplementation.

## 1. Introduction

Athletes are often perceived as exemplary of a healthy lifestyle, characterized by consistent engagement in physical activity, adherence to a balanced diet, and a proactive approach to health maintenance [1]. In recent years, there has been a notable increase in research and commercial interest directed toward the development of dietary supplements specifically tailored to the needs of physically active populations [2]. The rationale for supplementation among athletes is primarily based on the expectation that these products can enhance physical performance, optimize health outcomes, and expedite post-exercise recovery [3,4,5]. The most frequently consumed supplements include creatine, caffeine, isotonic beverages, vitamin D, energy bars, magnesium, and vitamin C [6,7].

Despite the common perception that athletes are generally better nourished than the average population [8], emerging evidence indicates that micronutrient deficiencies are prevalent within this demographic [9]. Among these, vitamin D deficiency appears particularly common [4]. The elevated physical workload experienced by athletes increases metabolic demands, rendering adequate dietary intake and targeted supplementation of deficient vitamins especially critical [10]. Nevertheless, the current dietary reference intakes for vitamins and minerals recommended for athletes are not differentiated from those for the general population [11].

A balanced diet plays a pivotal role in sustaining optimal athletic performance due to the diverse physiological functions of vitamins in the context of exercise [12,13,14,15,16]. These functions include antioxidant defense, regulation of energy metabolism, facilitation of blood coagulation, modulation of immune responses, promotion of tissue repair, and maintenance of bone mineralization [4,6,10,17]. In addition, adequate vitamin intake—whether achieved through diet or supplementation—has been shown to reduce fatigue in physically active individuals [17]. Conversely, inadequate vitamin supply is associated with an increased risk of musculoskeletal injuries [18], heightened susceptibility to acute illnesses [13,19], diminished sports performance [13], and delayed recovery following strenuous exercise [20].

Given the evidence that adequate vitamin intake exerts a positive influence on both exercise performance and post-exercise recovery, it is concerning that literature directly addressing the effects of vitamin supplementation in relation to specific sports disciplines remains scarce. Studies investigating the long-term effects of vitamin supplementation in athletes are minimal. Furthermore, methodological shortcomings are frequently observed, including reliance on retrospective and self-reported dietary assessments, as well as the inclusion of small sample sizes, which collectively constrain the reliability and external validity of findings [21,22,23,24].

Another significant limitation is that most existing reports lack practical applicability for coaches, sports nutritionists, and practitioners. Few studies provide discipline-specific, age-specific, or sex-specific supplementation guidelines, thereby limiting the translation of research into actionable strategies [25,26,27,28]. This represents a critical gap in the evidence base, as tailored recommendations are essential for optimizing supplementation protocols to meet the diverse needs of athletes participating in various sports and training regimens.

In response to these deficiencies, this narrative review aims to synthesize the available evidence from the past decade, with reference to earlier studies as necessary. The primary objective is to formulate specific, evidence-based recommendations that guide supplementation strategies for physically active individuals, thereby contributing to enhanced athletic performance, improved recovery, and overall health.

Finally, several limitations inherent to this review should be acknowledged. The narrative synthesis approach, although appropriate in light of the considerable heterogeneity of study designs, populations, and measured outcomes, carries an inherent risk of interpretation bias. Moreover, the inclusion of studies with varying methodological rigor may impact the strength and reliability of the conclusions presented.

In interpreting the evidence, we weighted conclusions according to methodological rigor—drawing stronger, more confident inferences from replicated randomized controlled trials (e.g., vitamins D and C), while applying intentionally cautious, conservative wording in sections where evidence is sparse, heterogeneous, or based primarily on mechanistic or observational studies (e.g., vitamins A and K).

## 2. Methods

*Databases and Search Dates:* We conducted structured searches in PubMed, ScienceDirect, PEDro, and the Cochrane Library, covering the period from 2010 to 2024, supplemented by earlier or later seminal works as necessary.

*Search Strings*: Search strings combined vitamin-specific and performance-related terms using Boolean operators:

(“vitamin A” OR “retinol” OR “retinoic acid”) OR (“vitamin B” OR “thiamine” OR “riboflavin” OR “niacin” OR “pyridoxine” OR “folate” OR “cobalamin”) OR (“vitamin C” OR “ascorbic acid”) OR (“vitamin D” OR “25(OH)D”) OR (“vitamin E” OR “tocopherol”) OR (“vitamin K” OR “phylloquinone” OR “menaquinone”) AND (“athletes” OR “sports” OR “exercise” OR “performance” OR “recovery” OR “injury prevention”).

*Eligibility*: The presented study utilized peer-reviewed human studies (including both athlete and non-athlete populations) that reported vitamin status, supplementation, or performance outcomes. Mechanistic and animal studies were considered only when directly relevant to underlying physiological pathways. Inclusion: Human studies (RCTs, cohort, cross-sectional, case–control) examining vitamin status, supplementation, or performance outcomes in athletes or active adults. Exclusion: Case reports, non-peer-reviewed materials, animal studies (except when elucidating physiological mechanisms), and studies combining vitamins with multiple ergogenic aids where vitamin effects were indiscernible. Population Handling: Data from non-athlete studies were included only when providing mechanistic insights relevant to metabolic or physiological pathways in sport; these are clearly labeled as extrapolations.

*Athlete vs. Non-Athlete Data*: When data derived from non-athlete populations were discussed, these were clearly labeled as extrapolations to provide mechanistic or contextual background, not direct evidence for athletes.

*Quality Appraisal:* As a narrative review, we did not apply formal systematic review tools; however, we performed a structured quality appraisal of included studies using the SANRA (Scale for the Assessment of Narrative Review Articles) criteria, focusing on the justification of the article’s importance, the comprehensiveness of the literature search, the level of evidence, and the balance of presentation.

*Critical Appraisal*: Study rigor and narrative balance were assessed using the SANRA criteria, evaluating (1) justification of topic importance; (2) clarity of aims; (3) comprehensive literature coverage; (4) transparent referencing; (5) scientific reasoning and balance; and (6) explicit statement of limitations. Each study was qualitatively graded for methodological clarity, population relevance, and reproducibility of findings. Table 1 provides an overview of the studies included in this review.

## 3. Water-Soluble Vitamins

### Vitamin C

The relationship between vitamin C supplementation and athletic performance has been a critical area of investigation and the subject of numerous literature reviews over the last forty years [29,30,31,32,33,34,35,36,37,38,39,40,41,42,43,44,45].

As a potent antioxidant, vitamin C plays a crucial role in mitigating oxidative stress that occurs during intense physical exercise [46]. Analysis of the recent literature revealed that the role of vitamin C extends beyond reducing oxidative damage. It is also integral in enhancing overall physical performance and recovery following strenuous activities. A recent study has indicated that athletes who participate in aerobic activities and consume vitamin C experience less muscle soreness and quickly return to baseline performance following physical effort [17]. In high-intensity training regimens that increase free radical production, supplementation with vitamin C can help support stronger training adaptations [47]. Patlar et al. [48] in their study on the effects of vitamin C in mitigating oxidative stress during rigorous training discovered that moderate-dose vitamin C supplementation (e.g., 300 mg/day) significantly prevented lipid peroxidation in athletes performing exhaustive exercises.

It was also shown that vitamin C supplementation can significantly decrease markers of oxidative stress during and after exercise when administered orally in a dose of 300 mg/day [48] or when administered orally for 14 days, 4 times a day, in a 500 mg dose [49]. Junaidi et al. [17] and Günalan et al. [7] confirmed that vitamin C helps reduce exercise-induced muscle damage.

Recent studies have confirmed that supplementation with vitamin C may also enhance the post-training recovery process in athletes by influencing both metabolic and inflammatory responses [48,49,50]. Furthermore, in a controlled study involving basketball players, decreased cortisol levels were observed in subjects supplemented with vitamin C at doses ranging from 0.25 to 1.0 g per day [51]. By lowering cortisol levels, vitamin C may improve subsequent performance during later training sessions, suggesting a direct benefit for athletes looking to maintain high levels of performance over time [50].

While the benefits of vitamin C are well-documented in endurance sports [29,52,53], its potential advantages also extend to other forms of athletic activity. For example, in combat sports, the antioxidant properties of vitamin C have been linked to improved recovery from muscle injuries and better overall training adaptations [54]. Chou et al. [50] demonstrated that short-term high-dose vitamin C supplementation significantly reduced muscle damage and inflammatory responses in athletes participating in intense Taekwondo competitions. These observations suggest that vitamin C supplementation may mitigate the severity of muscle injuries and the associated inflammatory response, which, in consequence, leads to improved recovery outcomes. A probable influence on tendinopathy recovery, a phenomenon accounting for a substantial part of all sports injuries and occupational disorders [55], has also been critically acclaimed in the study by Noriega-Gonzalez et al. [56] following supplementation with vitamin C.

Nevertheless, the latest findings on the efficacy of vitamin C supplementation are still debated. Some studies suggest that excessive antioxidant supplementation may hinder the positive adaptations to training by disrupting signaling pathways that facilitate beneficial cellular responses to oxidative stress [54,55]. Kim et al. [56] noted that while antioxidant supplementation can aid recovery, it may interfere with the physiological adaptations necessary for enhancing strength and endurance. It reinforces the recommendation by Wilson-Barnes et al. [25] that athletes should aim to achieve adequate vitamin intake through a balanced diet rather than solely through supplements.

While vitamin C can reduce oxidative damage [57], it may also dampen the body’s natural over-compensation mechanisms that typically occur after strenuous exercise [44,58]. Thus, moderation and timing of vitamin C intake are paramount for athletes aiming to maximize performance while reaping the benefits of this nutrient [43,50].

Furthermore, the form of vitamin C supplementation—i.e., through dietary sources or as an isolated supplement—can influence its efficacy [59]. Because whole food sources of vitamin C, such as fruits and vegetables, provide additional benefits derived from other phytochemicals and micronutrients that may enhance absorption and utilization [60]. It is a compelling rationale for athletes to focus on a well-rounded diet that includes rich sources of vitamin C, along with adequate protein and carbohydrates necessary for recovery [44,58].

In conclusion, vitamin C supplementation holds promise in supporting athletic performance, particularly by reducing muscle soreness and oxidative stress while enhancing recovery. However, athletes must strike a careful balance in their approach to supplementation to avoid potential negative impacts on exercise adaptations as observed by Nikolaidis et al. [61] and Shunchang et al. [7]. Future research should continue to explore optimal dosing strategies, the timing of supplementation relative to training sessions, and the effects of dietary sources of vitamin C on athletic performance outcomes. The benefits and risks of vitamin C supplementation are compiled in Table 2.

It is worth noting that since the body typically regulates vitamin C levels tightly, excreting excess amounts through urine, doses greater than 2 g per day may result in side effects, such as gastrointestinal disturbances (diarrhea, nausea, and abdominal cramps) [49]. Additionally, there is evidence that excessive vitamin C supplementation could interfere with the absorption of other nutrients and minerals, particularly copper and selenium, which can lead to complications in overall health and athletic performance [51]. Furthermore, individuals with certain health conditions, such as kidney disease, may be particularly at risk, as high levels of vitamin C can lead to the formation of kidney stones, especially oxalate stones, in susceptible individuals [54,74,75,76]. Individuals with glucose-6-phosphate dehydrogenase (G6PD) deficiency may experience hemolysis when exposed to high doses of vitamin C [77,78,79,80]. It was also demonstrated that ultra-high doses of vitamin C are associated with hemolytic events [74,81]. Moreover, in individuals with hemochromatosis or those with high iron stores, excessive vitamin C intake may exacerbate iron overload, potentially leading to toxicity. This risk is particularly concerning for patients with genetic predispositions to iron accumulation [81]. In the presence of transition metals like iron and copper, vitamin C can act as a prooxidant. This dual role raises concerns about its safety in high doses, as it may contribute to oxidative damage rather than prevent it [82].

Despite the abundance of data on vitamin C application in sports, the study suffers from methodological limitations. Thus, (1) the heterogeneity of study designs, populations, and outcome measures in vitamin C research presents significant challenges for evidence synthesis. (2) Analysis of the literature revealed that many studies employ different supplementation protocols, making direct comparisons difficult. (3) Additionally, the definition of” athletic populations” varies widely, from recreational exercisers to elite competitors, further complicating interpretation, and (4) the lack of standardized outcome measures is particularly problematic.

Analysis of the literature allowed for elucidation of the targeted supplementation protocol. Thus, the moderate-dose protocol (recommended for most athletes) encompasses a dosage of 200–500 mg daily, divided into two doses, i.e., a morning dose of 200–300 mg with breakfast and evening dose of 100–200 mg with dinner, with special consideration given to avoiding taking vitamin C immediately before or after key training sessions [83].

## 4. B Vitamins

The B-vitamin complex includes eight water-soluble vitamins—B1 (thiamine), B2 (riboflavin), B3 (niacin), B5 (pantothenic acid), B6 (pyridoxine), B7 (biotin), B9 (folate), and B12 (cobalamin). They are critical in sports and physical performance due to their roles in energy production [84], red blood cell synthesis [85], neurological function [86], and tissue repair [87]. Although each B vitamin has distinct physiological functions, they often work together as coenzymes in key metabolic pathways that support athletic performance and recovery [14,88,89,90,91].

It can be observed that thiamine (B1) is strongly linked to carbohydrate metabolism and aerobic energy production, thereby reducing fatigue by activating pyruvate dehydrogenase [88,92]. Riboflavin (B2) contributes to aerobic metabolism, reduces muscle pain, and accelerates recovery, as supported by double-blinded trials [92,93]. Niacin (B3) supports the formation of NAD/NADP coenzymes, which are essential for glycolysis and the citric acid cycle, with emerging links to the modulation of oxidative stress [94,95]. Pantothenic acid (B5), a precursor to coenzyme A, underpins fatty acid metabolism [96,97], while pyridoxine (B6) may enhance immune response and muscular endurance [98]. Biotin (B7) functions in carboxylation reactions essential for macronutrient metabolism [99], though evidence in athletes is limited. Folate (B9) supports amino acid metabolism and regulates homocysteine levels, influencing cardiovascular health and inflammation [100,101]. Vitamin B12 facilitates red blood cell synthesis and oxygen transport, while also supporting cognitive processing and reaction speed [88,102,103].

A recent study found that supplementation with vitamin B1 (thiamine) is associated with lower levels of pyruvate and lactic acid, which helps reduce fatigue during high-intensity exercise [88]. It suggests that thiamine can enhance performance by improving energy metabolism and delaying fatigue [104]. Because thiamine activates the pyruvate dehydrogenase complex, boosting glucose-to-energy conversion, it may benefit athletes in aerobic sports [105].

Its active form, thiamine pyrophosphate, plays a crucial role in carbohydrate metabolism during exercise [104]. It supports the role of thiamine in boosting aerobic metabolism and energy production [105]. A broader review supports the idea that adequate vitamin intake enhances muscle function, recovery, and athletic output [4].

However, thiamine deficiency can impair metabolism and negatively impact performance [106], leading to fatigue and reduced performance [107,108], particularly under physical stress, such as in combat sports [106].

A double-blinded, placebo-controlled trial on the influence of vitamin B2 on sports performance [94] suggested that riboflavin supplementation before and during prolonged running may reduce muscle pain and soreness during and after exercise, as well as enhance early functional recovery after the workout. This observation was complemented by a randomized, placebo-controlled double-blinded trial conducted by Kent et al. [93], who showed that the group consuming riboflavin had a significantly shorter recovery time after a bout, 9.9 days versus 22.2 days in the placebo group (*p* < 0.05). It has also been shown that riboflavin enhances aerobic power and recovery [107]. Athletes generally maintain adequate B2 levels, likely due to higher dietary intake [108].

Vitamin B3, also known as niacin or nicotinic acid, plays several critical roles in athletic performance by modulating energy metabolism and neurological function. Vitamin B3 (niacin) enhances athletic performance by modulating energy metabolism and neurological function through the formation of coenzymes NAD and NADP, which are vital in glycolysis and the citric acid cycle [88]. While some research links niacin to reduced oxidative stress and improved muscle recovery, evidence in athletes remains under evaluation [94,95]. Ghazzawi et al. showed that adequate niacin may help sustain performance by maintaining energy reserves and supporting cardiovascular health [109]. In high-stress environments, it could also support neurotransmitter activity and mental stamina [110]. However, the recent study shows that vitamins B1, B2, and B3 do not consistently produce ergogenic effects [111].

Pantothenic acid (B5) is essential in energy metabolism [96], fatty acid synthesis [112], and coenzyme production [113]. As a coenzyme A precursor, B5 plays a role in aerobic energy metabolism [97]. However, it does not improve aerobic performance [114]. Combined with other B vitamins, it may support endurance and recovery; however, further studies are needed [115].

Data on the applicability of B6 (pyridoxine) in sports is scarce. However, a recent study showed that it may modulate the immune response and, when combined with other B vitamins, improve agility and muscular endurance [98]. Manore et al. [116] showed that women are prone to a decline in pyridoxine levels during periods of dieting and exercise.

Little is known about biotin (B7) in athletic contexts, but in general metabolism, it acts as a CO_2_ carrier in carboxylation reactions that are vital for the synthesis of fatty acids, amino acids, and carbohydrates [99]. In animal models, B7 has been shown to improve traits under oxidative stress [117].

Folate (B9) is crucial for amino acid metabolism [118]. Its deficiency elevates homocysteine [100] and increases the risk of cardiovascular disease [119,120]. In athletes, it affects endurance, making folate vital to training and peak performance [109]. It may also lower C-reactive protein, reducing inflammation [101]. Studies have linked higher folate intake with improved performance and recovery [121,122], particularly in female elite athletes [123]. It has been shown that vegetarian and vegan athletes are at risk of folate deficiency [103,120].

The synthesis of red blood cells is a crucial process facilitated by vitamin B12, which serves as a coenzyme in the conversion of homocysteine to methionine, a vital step in DNA synthesis and cellular replication [102]. A deficiency in vitamin B12 can lead to megaloblastic anemia, characterized by larger-than-normal red blood cells that are ineffective in transporting oxygen [124]. For athletes, this may lead to decreased endurance and increased fatigue. Some studies indicate that a well-maintained level of vitamin B12 is essential for preserving optimal endurance capabilities during exercise [109].

Vitamin B12 supports red blood cell formation by converting homocysteine to methionine, a step required for DNA synthesis and cell replication [102]. Deficiency leads to megaloblastic anemia, impairing oxygen transport and reducing endurance capability [124]. Proper B12 levels help maintain stamina and recovery by supporting fatty acid and amino acid metabolism [109]. Adequate B12 reduces lactate build-up during intense training [4]. Vegetarians and vegans face a higher risk of B12 deficiency [105,124], necessitating careful monitoring and potential supplementation [88]. Supplementation with B12 helps maintain” appropriate” levels, critical for sustaining metabolism, endurance, and recovery during sports bouts [103]. B12 is also essential for cognition, aiding processing speed and decision-making—crucial for competitive athletes [88,103]. Reduced vitamin B12 levels could potentially lead to declines in mental performance, affecting reaction times and decision-making skills—the latter being vital for competitive athletes, especially in fast-paced sports [103].

A study by Lee et al. [88] found that 28 days of B-complex supplementation (including B1, B2, B6, and B12) improved endurance and reduced fatigue without any adverse effects. Nevertheless, other studies show that B1, B2, and B3 may not yield ergogenic benefits unless thiamine derivatives are involved [111]. Thiamin, riboflavin, and B6 remain key to energy metabolism, which is vital for athletes [125,126].

A study involving overweight and obese men found that B-complex supplementation combined with sports improved physical activity and body composition, reducing fat and increasing lean mass [114]. These findings suggest that targeted vitamin B supplementation, combined with structured training, can improve fitness outcomes. However, vitamin B supplementation is not necessary for athletes with balanced diets, and excessive intake may offer no benefits and should be avoided [114,127]. Nevertheless, athletes with poor diets may derive the most benefit from vitamin B complex supplementation [125]. The summary of the recent findings is compiled in Table 3.

The literature on the relationship between B vitamins and metabolic pathways is illustrated in the figure in Section 5.2. It can be observed that thiamine (B1) plays a crucial role in glycolysis and the TCA cycle through its active form, thiamine pyrophosphate, which activates the pyruvate dehydrogenase complex, thereby enhancing glucose-to-energy conversion [88,104,105]. Riboflavin [111] is central to aerobic energy metabolism via its coenzymes FAD and FMN, which are integral to the TCA cycle and fatty acid β-oxidation [92,107]. Niacin [111] contributes significantly to NAD/NADP formation, which is essential for redox reactions in glycolysis, the TCA cycle, and oxidative phosphorylation [94,95]. Pantothenic acid [96] is the precursor for coenzyme A, a fundamental cofactor in both fatty acid oxidation and the TCA cycle [96,97]. Pyridoxine [4] participates indirectly in energy metabolism by supporting amino acid transamination and gluconeogenesis [125,126]. Biotin [117] is a cofactor for carboxylase enzymes, enabling key reactions in fatty acid synthesis and gluconeogenesis [99]. Folate [118] has minimal direct involvement in core energy pathways but is crucial for amino acid metabolism, indirectly supporting energy availability [100]. Vitamin B12 is required for odd-chain fatty acid metabolism and methylmalonyl-CoA conversion to succinyl-CoA, linking it to the TCA cycle [102].

Analysis of the current literature on cross-correlations between B vitamins supplementation and sports activities unfolded the limitations including: (1) heterogeneity in supplementation protocols and dosages, (2) variation in study populations and athletic disciplines, and (3) limited long-term safety data for high-dose supplementation and the evidence gaps on (1) optimal dosing for specific sports and training phases, (2) individual variation in B vitamin requirements, (3) interaction effects between different B vitamins, and (4) long-term performance and health outcomes.

Based on successful intervention studies, effective dosing is provided in Table 4:

## 5. Vitamin E

**Figure 1:**
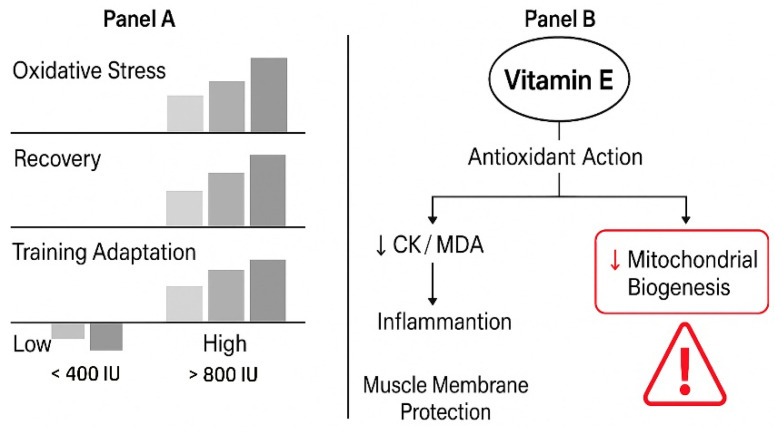
Dose–response and function outcomes of vitamin E supplementation in athletes [43,52,138,139,140]. Panel (**A**): Cross-correlations between the levels of vitamin E and the levels of oxidative stress, muscular recovery, and training adaptation. Panel (**B**): Antioxidant action of vitamin E and its influence on inflammation processes and mitochondrial biogenesis.

Among the early studies on the effect of vitamin E supplementation on physiological parameters associated with physical training was the report by Helgheim et al. [137]. In a study involving 26 trained and untrained individuals aged 19–24, the levels of serum enzymes were assessed after heavy exercise in response to d-α-tocopherol supplementation. After a six-week medication period (300 mg daily; 450 UI), the serum concentration of d-α-tocopherol increased from 12.7 to 19.6 mg/mL in the vitamin E group, while in the placebo group, the value remained unchanged. Participants of the study were subjected to muscular work involving either trained or untrained muscle groups. Serum levels of creatine kinase (CK), aspartate aminotransferase (ASAT), and lactate dehydrogenase (LD) were measured before exercise and at various time points post-exercise. In individuals exercising with trained muscles, serum enzyme levels showed only minor, statistically insignificant elevations, with no differences observed between the vitamin E and placebo groups (Figure 1).

In contrast, exercise involving primarily untrained muscles led to a significant increase in serum CK activity, alongside notable elevations in ASAT and LD [141,142,143]. Again, no differences were detected between the vitamin E and placebo groups [144,145,146]. Isoenzyme analysis revealed that the rise in CK was attributable to the CK-MM isoform, suggesting that the primary source of enzyme release was striated skeletal muscle [147,148]. These findings indicate that vitamin E supplementation does not influence post-exercise increases in serum enzyme concentrations [144].

Recent research has recognized vitamin E as a potent antioxidant, playing an essential role in protecting cellular membranes from oxidative damage during intense physical activity [149,150,151]. Research has shown that supplementation with vitamin E can reduce exercise-induced oxidative stress markers, such as malondialdehyde and creatine kinase levels, aiding in muscle recovery [152,153]. Specifically, vitamin E has been associated with reduced muscle damage and inflammation in response to repeated strenuous exercises, underscoring its importance for athletes [154,155].

Studies suggest that athletes, particularly those engaged in endurance activities, may have higher demands for antioxidants due to the increased production of reactive oxygen species (ROS) during exercise [145,156]. High-dose vitamin E supplementation has been shown to have protective effects against muscle injury and inflammatory responses in athletes undergoing rigorous training regimens [157,158,159]. Furthermore, a study on elite Indian cyclists supported the hypothesis that vitamin E could decrease oxidative markers resulting from endurance training [159].

Conversely, recent literature has indicated that high doses of antioxidant vitamins, including vitamin E, may blunt the physiological adaptations to endurance training, such as mitochondrial biogenesis and protein synthesis essential for performance improvement [139,160]. Notably, studies have reported that supplementation with antioxidants might impair the beneficial adaptations that typically occur with regular exercise, leading to a paradoxical effect that could negate potential endurance benefits [63,161]. It suggests that while vitamin E supplementation may enhance recovery and reduce acute muscle damage, it might concurrently interfere with long-term adaptations necessary for sustained athletic improvement.

Moreover, the findings of the last decade confirm the role of vitamin E in mitigating oxidative stress induced by various forms of physical exertion, particularly among athletes. The recent studies observed that oxidative stress arises in response to the production of reactive oxygen species (ROS) during intense exercise, which can lead to muscle damage and impaired athletic performance if not adequately managed [43,162]. In a clinical trial, egg supplementation enriched with *n*-3 polyunsaturated fatty acids and antioxidants, including vitamin E, was shown to enhance microvascular adaptation and reduce oxidative stress during strenuous physical exercise in male athletes [163]. It suggests that maintaining higher levels of vitamin E may be beneficial in preserving muscle integrity during high-intensity workouts [164]. Furthermore, evidence from studies demonstrates that vitamin E, particularly in combination with vitamin C, can diminish markers of muscle damage, such as creatine kinase levels, following intense exercise [165,166].

However, findings concerning the efficacy of vitamin E supplementation are not without controversy (Table 5). Some studies have reported that antioxidant supplementation, including vitamin E, may hinder muscular adaptations from training. For example, one study observed that vitamin E had detrimental effects on the proteome response to training, suggesting that excessive antioxidant intake could blunt the positive adaptations expected from endurance training [167]. It indicates a complex interaction where the timing and dosage of vitamin E supplementation are crucial; while it might protect against oxidative damage, it could also interfere with the body’s natural adaptive processes when overconsumed.

Moreover, the role of vitamin E in sports should also consider dietary sources and the overall nutritional strategy employed by athletes [16,168,169]. While supplementation can be beneficial, a balanced diet rich in antioxidants, including natural sources of vitamin E, may provide a more effective means of managing oxidative stress [170,171]. Several studies emphasize the importance of athletes meeting their overall nutritional needs to optimize their performance [109,172].

**Table 5:** The summary of key findings on vitamin E and athletic performance. (The arrows indicate elicited physiological response).

Study/Year	Participants	Intervention	Main Outcomes	Conclusion
Helgheim et al., 1979 [137]	26 trained & untrained men, 19–24 years of age	300 mg/day d-α-tocopherol for 6 weeks	↑ serum vitamin E in supplement group; no effect on CK, ASAT, LD changes post-exercise (trained/untrained muscles)	Vitamin E did not alter post-exercise enzyme increases
Sureda et al., 2008; Bojanić et al., 2013 [152,153]	Various athlete groups	Vitamin E supplementation	↓ oxidative stress markers (MDA, CK), ↑ recovery	Supports antioxidant protection during intense exercise
Chou et al., 2018; de la Puente Yagüe et al., 2020 [154,155]	Athletes under repeated strenuous exercise	Vitamin E supplementation	↓ muscle damage & inflammation	Beneficial for recovery
Yusni et al., 2019; Bădău et al., 2018; Chhavi et al., 2009 [157,158,159]	Endurance athletes	High-dose vitamin E	↓ oxidative markers, ↓ muscle injury	Potential protective role in endurance training
Higgins et al., 2020; Rothschild et al., 2019 [139,160]	Endurance athletes	High-dose antioxidants (including vitamin E)	↓ mitochondrial biogenesis, ↓ protein synthesis	High doses may blunt long-term adaptations
Kolar et al., 2023 [163]	Male athletes	Egg supplementation enriched with *n*-3 PUFA + antioxidants (vitamin E)	↑ microvascular adaptation, ↓ oxidative stress	The combined nutrition approach is effective
Martínez-Ferrán et al., 2022; Koohkan et al., 2023 [165,166]	Athletes in high-intensity training	Vitamin E + vitamin C	↓ CK, ↓ muscle damage	Synergistic antioxidant effects
Wyckelsma et al., 2025 [167]	Athletes	Vitamin E supplementation	Negative impact on the proteome response to training	Potential interference with adaptation
Dobrowolski et al., 2024; Ghazzawi et al., 2023 [109,172]	General athletic population	Dietary vitamin E	A balanced diet with antioxidants supports performance	Food sources preferred over high-dose supplements

The limitations of the reviewed study encompass the following: (1) heterogeneity in study designs and populations limited meta-analytic approaches, (2) most studies had relatively short follow-up periods, (3) baseline vitamin E status was rarely assessed, and (4) publication bias may favor studies showing positive results.

Literature analysis revealed the following dosage and timing considerations [114,139,154,173]. Thus, studies employed varying dosages ranging from 100 to 1000 IU daily: (1) 100–400 IU daily: most commonly studied range with minimal benefits; (2) 400–800 IU daily: some positive effects in combination studies; and (3) <800 IU daily: limited studies with no additional benefits.

Although the literature analysis allows for the establishment of specific doses of vitamins C and E, the recent consensus in sports nutrition emphasizes caution with antioxidant supplementation, as excessive or peri-exercise intake of these vitamins may attenuate key adaptive responses to training, including mitochondrial biogenesis and redox signaling [63,174,175,176,177]. Therefore, rather than fixed dosing protocols, a conditional decision pathway is proposed: (a) confirm deficiency or clinical indication through dietary assessment or biochemical testing before supplementation; (b) consider the training phase, avoiding antioxidant supplementation during adaptation-focused blocks (e.g., build or overload) and restricting use to recovery or taper phases if needed; (c) prioritize a food-first strategy, emphasizing antioxidant-rich fruits, vegetables, and whole foods; and (d) if supplementation is deemed necessary, avoid high-dose or peri-exercise administration (e.g., ≥500 mg vitamin C or ≥400 IU vitamin E). These recommendations constitute conditional guidance with low certainty, reflecting heterogeneity in existing evidence and the absence of athlete-specific randomized controlled trials [156,176,178,179,180,181,182,183]. This individualized, phase-specific approach better aligns with current evidence linking antioxidant balance—not maximal intake—to optimal training adaptation and recovery.

### 5.1. Vitamin A

**Figure 2:**
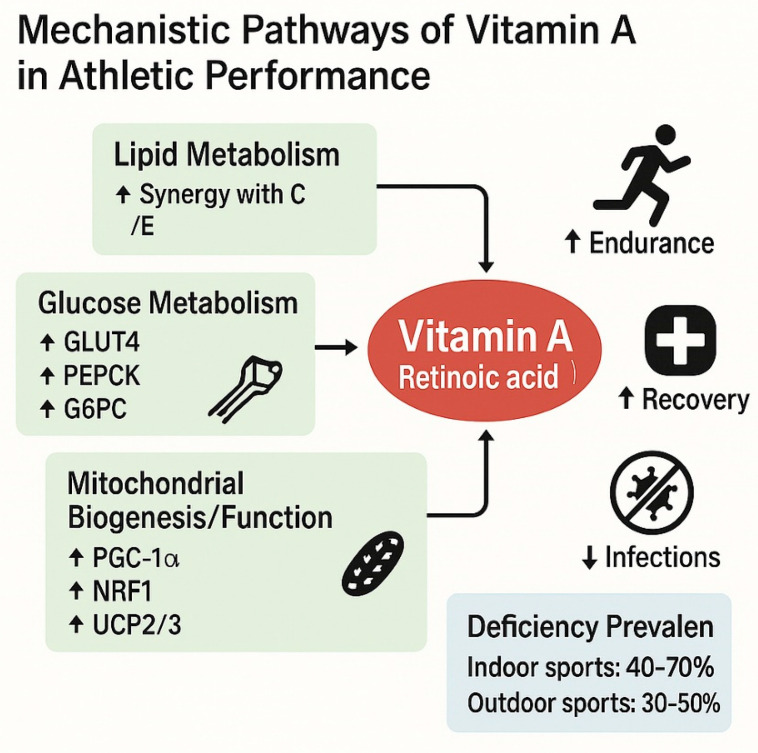
Literature-derived mechanistic pathways of vitamin A as a function of sports activity [184,185,186,187].

The role of vitamin A in sports performance has gained increased attention due to its multifaceted effects on metabolism, immune function, and overall health (Figure 2). Vitamin A, which includes retinol, is essential for various physiological functions critical to athletic performance. Generally, it influences energy metabolism, reduces oxidative stress, enhances immune function, and promotes recovery.

Vitamin A is primarily known for its role in vision and epithelial integrity, where it functions as a modulator of gene expression through its active metabolite, retinoic acid [188]. Altogether, vitamin A affects muscle repair, mitochondrial biogenesis, and protein synthesis [189]. Moreover, its immunomodulatory effects support the immune system under the stress of intense physical activity, potentially reducing susceptibility to infections in athletes [190,191].

Vitamin A also plays a noteworthy role in the metabolism of macronutrients. The active metabolite of vitamin A, all-trans-retinoic acid (ATRA), acts as a ligand for nuclear receptors—retinoid X receptors (RXRs) and retinoic acid receptors (RARs), and regulates the transcription of a variety of genes involved in metabolic pathways [192,193,194]. These receptors are known to form heterodimers with other nuclear receptors such as PPARs (peroxisome proliferator-activated receptors) and thyroid hormone receptors, which are directly involved in lipid oxidation, glucose homeostasis, and mitochondrial function [195].

In lipid metabolism, retinoic acid enhances fatty acid oxidation and suppresses lipogenesis in the liver and adipose tissue by controlling the expression of genes such as CPT1 (carnitine palmitoyltransferase 1) and SREBP-1c [192].

In carbohydrate metabolism, vitamin A status influences insulin sensitivity and glucose transport; deficiencies have been linked to impaired gluconeogenesis and dysregulated blood glucose levels [196]. Additionally, vitamin A plays a role in protein metabolism through its effects on cellular differentiation and muscle protein synthesis, partly by interacting with growth-related signaling pathways [197].

During physical exercise, several metabolic pathways that utilize micronutrients, including vitamin A, are activated, facilitating efficient energy production [198,199,200].

Since the body’s demand for ATP increases substantially, requiring enhanced activity of metabolic pathways such as glycolysis, β-oxidation, the tricarboxylic acid (TCA) cycle, and oxidative phosphorylation, vitamin A contributes to these processes through its regulatory effects on gene expression and its antioxidant and immunomodulatory properties [84,201].

Vitamin A also has profound effects on mitochondrial function and biogenesis. It upregulates peroxisome proliferator-activated receptor gamma coactivator 1-alpha (PGC-1α), a master regulator of mitochondrial biogenesis, either directly or through interactions with estrogen-related receptors (ERRα) and PPARδ [189]. Additionally, it stimulates the expression of uncoupling proteins (UCP2, UCP3) and nuclear respiratory factors (NRF1, NRF2), contributing to enhanced mitochondrial oxidative capacity and energy efficiency [194].

In the regulation of glucose metabolism, vitamin A influences both glucose uptake and hepatic glucose production [202,203,204,205]. It increases the expression of GLUT4 (SLC2A4) in skeletal muscle and adipose tissue, thereby improving insulin sensitivity and peripheral glucose uptake [206]. Simultaneously, it suppresses gluconeogenic enzymes such as glucose-6-phosphatase (G6PC) and phosphoenolpyruvate carboxykinase (PEPCK), reducing hepatic glucose output [193]. Furthermore, vitamin A induces pyruvate dehydrogenase kinase 4 (PDK4), which shifts the substrate preference towards fatty acids and away from glucose oxidation, promoting metabolic flexibility during fasting or energy-demanding states [173].

These synergistic effects are crucial during endurance and resistance exercises, where efficient substrate utilization is essential for maintaining performance. Moreover, retinoic acid enhances mitochondrial biogenesis and fatty acid oxidation via its interaction with nuclear receptors such as PPARs and RXRs, which coordinate the transcription of genes like PGC-1α, a master regulator of mitochondrial metabolism [207].

Since exercise also increases the generation of reactive oxygen species (ROS), vitamin A stabilizes cell membranes and interacts with antioxidants, such as vitamins C and E, to reduce oxidative stress [190]. Furthermore, retinol, as an antioxidant, can counteract the oxidative damage caused by free radicals appearing during stressful physical exertion [208,209]. There is some evidence suggesting that adequate vitamin A levels may contribute to improved recovery and performance outcomes [122], but more targeted research is necessary to confirm these links. In summary, this antioxidant defense is crucial for protecting mitochondria and muscle cells from exercise-induced damage and fatigue.

A recent study revealed that athletes, particularly those engaging in high-intensity training, should ensure their diet includes sufficient vitamin A intake to meet the elevated metabolic demands imposed by their training regimens [13,210]. Insufficient vitamin A may lead to impaired energy metabolism, which can impact an athlete’s endurance and performance capabilities [109,153].

The role of vitamin A in immune function is equally critical for athletes. Mechanistic and animal studies have demonstrated that a weakened immune system can lead to increased susceptibility to illnesses, resulting in frequent absences from training and competition [28,94,211]. Research indicates that higher intakes of vitamin A contribute to enhanced immune response, particularly in athletes subjected to rigorous training regimens that may stress their immune systems [109]. Adequate vitamin A levels can help prevent infections and illnesses, allowing athletes to maintain consistent training schedules and ultimately enhance their performance.

Furthermore, vitamin A influences physiological functions related to muscle recovery and post-exercise adaptation [212]. The need for athletes to consume a well-rounded diet sufficient in all essential micronutrients, including vitamin A, to ensure optimal muscle recovery was recently confirmed [4,109]. Moreover, some studies have suggested that athletes meeting their recommended vitamin A intake may exhibit better recovery profiles, which is crucial for training adaptations and improved performance across competitive events [208,213]. The summary of all the above-mentioned findings is provided in Table 6.

The findings of the last 10 years on the subject revealed that vitamin A supplementation influences various physiological parameters among handball players, impacting oxidative balance, which may enhance athletic performance [158]. This finding aligns with other research highlighting that micronutrients, including vitamin A, are integral to recovery and athletic efficacy due to their roles in metabolic pathways and cellular functions critical during physical stress [109]. Notably, athletes undergoing intense training often experience increased oxidative stress; therefore, antioxidants, such as vitamin A, can mitigate these effects and aid recovery [4].

Furthermore, the importance of adequate micronutrient intake, including vitamin A, in athletes’ diets cannot be overstated. Nutritional deficiencies in elite athletes have been documented, with studies indicating that many athletes do not meet the recommended intakes of essential vitamins, including vitamin A [228,229]. This deficiency may adversely affect performance, recovery, and immune response, underscoring the need for tailored dietary strategies to meet the heightened needs of active individuals [213]. It is essential to ensure diets are balanced and that supplementation is considered where dietary intake may be insufficient.

Additionally, Chen and Liu [228] discuss how vitamins and minerals can influence the performance capabilities of athletes engaged in various sports, emphasizing that maintaining adequate micronutrient intake is crucial for optimal physiological functioning and performance [230]. A systematic review by Cruz et al. supports this view, indicating that training adaptations necessitate increased micronutrient consumption for adequate metabolic support [231].

Incorporating vitamin A into nutritional strategies for athletes could be a proactive measure, not only for performance enhancement but also for optimizing recovery and ensuring long-term health. This perspective is supported by the increasing recognition of personalized nutrition approaches in athletic preparation, which advocate for individual dietary assessments to meet specific nutrient needs based on training intensity and volume [232].

The evidence base for vitamin A suffers from several critical limitations: (1) the minimal number of controlled intervention studies specifically examining Vitamin A supplementation in athletes, (2) inconsistent dosing protocols and outcome measures across studies, (3) a lack of sport-specific research examining performance outcomes, (4) the absence of studies examining bone health outcomes despite Vitamin A’s known role in bone metabolism, and (5) limited investigation of muscle function parameters. Furthermore, current research fails to address key questions regarding optimal dosing, timing, sport-specific applications, and long-term safety considerations for Vitamin A supplementation in athletic populations.

Moreover, the evidence for the effects of Vitamin A supplementation on athletic performance is minimal and contradictory. While some research suggests potential antioxidant benefits, other studies indicate possible adverse effects. A concerning finding from animal research showed that Vitamin A supplementation (2000 IU/kg) in rats subjected to aerobic exercise actually enhanced oxidative stress in lung tissues and impaired exercise-induced adaptations of antioxidant enzymes [233]. It suggests that high-dose supplementation may interfere with beneficial training adaptations.

### 5.2. Vitamin D

**Figure 3:**
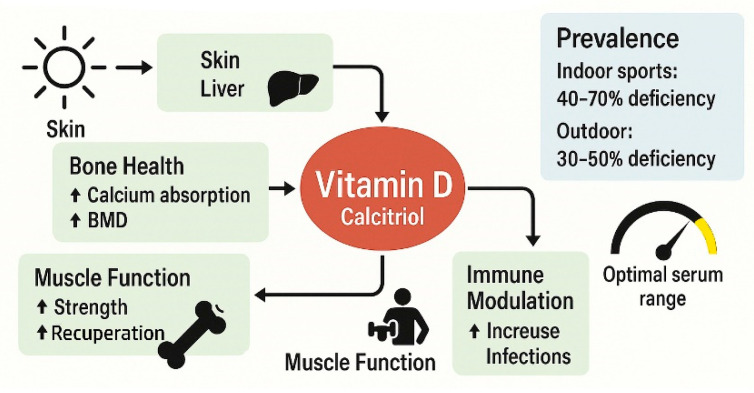
The role of vitamin D in musculoskeletal performance and injury prevention [12,45,236,237,238,239,240,241].

The application of vitamin D in the field of sports medicine has garnered attention in recent years, particularly in relation to athletic performance, musculoskeletal health, and injury prevention. Vitamin D is essential for various physiological functions, including muscle function [234] and bone metabolism [235], which are crucial for optimal athletic performance [155]. Current research highlights the benefits of vitamin D supplementation for athletes, leading to strategies that aim to enhance their overall health and performance (Figure 3).

Current evidence suggests that vitamin D supplementation in athletes has mixed effects on performance, with some benefits for aerobic capacity and anaerobic power, but a limited impact on muscle strength. Deficiency is prevalent (40–70% of athletes), particularly in winter and indoor sports, with optimal dosing protocols still unclear and safety concerns minimal at recommended doses [43,45,236,240].

Thus, it has been shown that due to limited sun exposure, particularly in indoor sports and during winter months, athletes obtain suboptimal levels of vitamin D [242,243,244]. This insufficiency is concerning, as vitamin D is integral to calcium absorption, which is essential for maintaining bone density and preventing stress fractures—a common injury among athletes [245,246]. Supplementation has been recommended as a means to normalize serum vitamin D levels, with studies suggesting that such interferences can lead to improvements in musculoskeletal health and performance metrics [241,245]. Indoor sports (gymnastics, swimming, basketball) show the highest deficiency rates, while outdoor sports demonstrate lower but still significant deficiency rates (30–50%) [247].

Systematic reviews highlight the positive impact of vitamin D supplementation on lower-body muscle strength, suggesting that athletes may experience gains in power and endurance following supplementation [236,241,248]. Additionally, vitamin D is thought to aid muscle recovery after exercise-induced damage, underscoring its role in not only injury prevention but also recovery processes [249,250]. It has been documented that athletes with adequate intake of calcium and vitamin D have a reduced risk of musculoskeletal issues and exhibit better recovery from intensive training sessions [251,252].

However, meta-analysis of 11 randomized controlled trials involving 436 athletes found no statistically significant effect of vitamin D supplementation on maximum strength and power for baseline serum 25(OH)D concentrations of <75 nmol/L [253].

These studies complement the findings of Frank et al. [254,255], who demonstrated that athletes with vitamin D deficiency exhibit higher rates of musculoskeletal injuries, with stress fractures being the specific injury type most commonly associated with deficiency. Moreover, recovery time from muscle strains was prolonged in vitamin D-deficient athletes [250,256].

Deficiencies in vitamin D have been associated with an increased risk of injuries, such as stress fractures and muscle strains, particularly in high-impact and endurance sports [245,257]. Studies focusing on collegiate athletes have shown that those with adequate vitamin D levels report fewer injuries and enhanced overall physical performance, suggesting a protective effect attributed to this micronutrient [244,258]. Therefore, focusing on vitamin D sufficiency may be a crucial aspect of injury prevention strategies in sports medicine [246,259]. It has also been observed that specific injury types associated with vitamin D deficiency include stress fractures and muscle strains, as well as prolonged recovery times from injuries [255].

Research suggests that adequate levels of vitamin D can improve muscular strength and functional performance. For example, a systematic review highlights the positive impact of vitamin D supplementation on lower-body muscle strength, suggesting that athletes may experience gains in power and endurance following supplementation [236,241,248]. Additionally, vitamin D is thought to aid muscle recovery after exercise-induced damage, underscoring its role in not only injury prevention but also recovery processes [249,250]. Moreover, an updated meta-analysis of 10 RCTs (encompassing 318 athletes) demonstrated significant increases in quadriceps contraction strength (SMD 0.57, 95% CI: 0.04–1.11, *p* = 0.04) [236].

Some studies suggest potential benefits for aerobic capacity and anaerobic power, particularly in vitamin D-deficient athletes [260], and there is limited evidence that suggests potential improvements in peak power output, with effects potentially more pronounced in explosive power activities [57]

An analysis of the recent literature unfolded cross-correlations between vitamin D supplementation and bone mineral density (BMD) in athletes. Thus, one study demonstrated significant improvements in bone mineral density (BMD) compared to the control group (*p* = 0.02) [252]. However, the other, performed on Singaporean athletes with sufficient vitamin D levels, showed significantly higher BMD compared to deficient athletes (*p* = 0.01) [22]

Nutritional strategies incorporating vitamin D should consider its interaction with other essential components, such as calcium and magnesium, both of which are crucial for bone health [245]. Consequently, dietary management strategies focusing on these nutrients, either through natural sources or supplements, are recommended in conjunction with routine vitamin D supplementation. However, when considering vitamin D supplementation, geographical latitude should also be taken into account, as it significantly influences the prevalence of deficiency [25].

Currently observed limitations of the study on vitamin D supplementation in sports include: (1) mixed evidence for performance enhancement and (2) baseline status as a critical determinant [45,237,243,261,262].

Target serum levels encompass the following values: optimal range for athletes: 75–125 nmol/L (30–50 ng/mL), deficient: <50 nmol/L (<20 ng/mL), insufficient: 50–75 nmol/L (20–30 ng/mL) and sufficient: >75 nmol/L (>30 ng/mL) [240,249,263,264]. The key findings on vitamin D application in sport are compiled in Table 7.

While vitamin D supplementation shows limited evidence for direct performance enhancement in athletes with adequate vitamin D status, it provides clear benefits for injury prevention and bone health maintenance, particularly in the 40–70% of athletes who are deficient, making baseline 25(OH)D concentration the critical effect-modifier that determines supplementation outcomes.

### 5.3. Vitamin K

Vitamin K supplementation in sports nutrition represents an emerging but understudied area of research. While vitamin K is well-established for its roles in blood coagulation and bone metabolism, its potential applications in athletic performance and recovery remain largely unexplored [266].

Vitamin K plays a crucial role in bone metabolism, primarily through the carboxylation of osteocalcin, a protein that is closely linked to bone strength and mineralization [267]. This relationship is particularly relevant for female athletes, as vitamin K deficiency can lead to lower bone mineral density and an increased risk of fractures [268,269]. Ishizu et al. [269] noted that dietary education to improve vitamin K and calcium intake is essential for young female athletes to maintain bone health, emphasizing the significant role these nutrients play in mitigating the risk of osteoporosis later in life. Furthermore, Yan et al. [88] indicated that dietary vitamin K can reduce systemic inflammation by lowering levels of pro-inflammatory cytokines, which can be detrimental to athletic performance and overall health.

**Figure 4:**
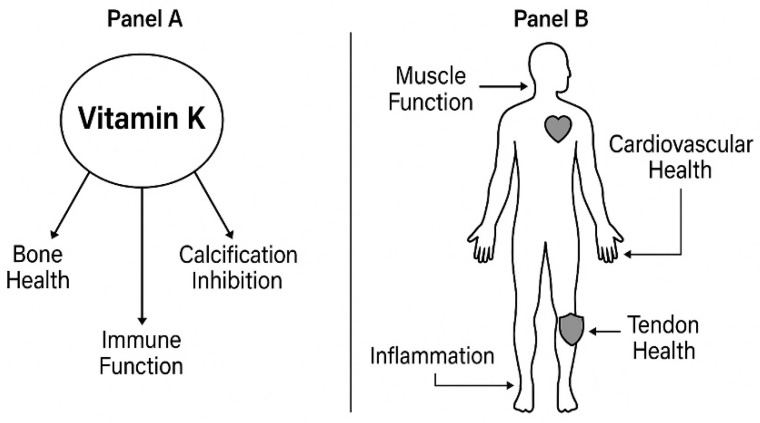
Vitamin K’s emerging roles in athletic health [19,270,271,272,273,274].

Besides its role in bone health, vitamin K may also play a role in muscle function (Figure 4). Research by Alonso et al. [268] suggests that higher vitamin K levels are associated with improved skeletal muscle function and might enhance muscle recovery following exercise. However, it is essential to note that while observational studies suggest these associations, interventional studies on vitamin K supplementation have shown conflicting results regarding improvements in muscle strength [56].

Vitamin K2’s cardiovascular effects are mediated through activation of matrix Gla protein, an anti-calcific protein [275]. Carboxylated matrix Gla protein effectively protects blood vessels and prevents calcification within the vascular wall [275].

Vitamin K’s anti-inflammatory properties may also help mitigate muscle damage associated with intense physical activity. It has been suggested that vitamin K may help prevent the inflammatory responses typically observed in athletes after exercise, potentially aiding in faster recovery and improved performance in subsequent training sessions [276]. Moreover, Dahlquist et al. identified vitamin K as a factor in the regulation of hepcidin, a hormone pivotal in iron metabolism, which is vital for endurance athletes susceptible to iron deficiency [277,278]. Given that iron is essential for oxygen transport and muscle function, adequate vitamin K levels may indirectly support athletic performance through improved iron status.

Ultimately, the interplay between vitamin K and other micronutrients, particularly vitamin D, underscores the complexity of nutrient interactions required for optimal athletic performance. The combination of vitamins D and K has been shown to influence muscle function and bone density, suggesting that these nutrients work synergistically to support physical health in athletes [277,279]. Therefore, ensuring adequate intake of both vitamins might be critical for athletes aiming to enhance their performance and recovery.

The limitations of the study on cross-correlations between vitamin K supplementation and sports can be categorized as follows: (1) lack of performance data: no completed studies demonstrate performance benefits in athletes; (2) dosing protocols: no established dosing guidelines for athletic populations; (3) timing strategies: no research on optimal timing of supplementation relative to training or competition; (4) safety in athletes: limited safety data for doses and durations relevant to sports applications; (5) mechanistic understanding: unclear how vitamin K’s known physiological roles translate to athletic benefits, and (6) population-specific effects: no data on how effects may vary by sport, training status, or demographic factors.

General recommendations for the specific form of vitamin K include the following: vitamin K1 (phylloquinone)—20 mcg/day for men, 90 mcg/day for women; vitamin K2 (menaquinone); MK-4 (synthetic): typically 45 mg/day in therapeutic application; MK-7 (natural): 100–200 mcg/day in most studies [280,281,282,283,284,285].

## 6. Summary

This review synthesizes findings from the past decade on the physiological impacts of vitamin supplementation in athletes, with a focus on both water-soluble (B vitamins and vitamin C) and fat-soluble (vitamins A, D, E, and K) compounds. High-intensity endurance sports are closely associated with increased oxidative stress and inflammatory responses. Antioxidant vitamins such as C, E, and A play a protective role by mitigating oxidative damage in skeletal, cardiac, and bone tissues. Vitamin E stabilizes cell membranes, vitamin C reduces serum cortisol, and vitamin K downregulates pro-inflammatory cytokines—collectively contributing to reduced inflammation and enhanced performance. Vitamin A further supports immune modulation and infection prevention, promoting training consistency.

In conclusion, vitamin C supplementation shows consistent benefits for reducing oxidative stress, muscle soreness, and cortisol levels, particularly at moderate daily doses. However, evidence for performance enhancement is inconsistent, and excessive intake may blunt adaptive responses. Overall, there is low to moderate certainty that vitamin C benefits recovery and immune defense in athletes, and low certainty for direct performance improvement. Food-first strategies are preferred, with supplementation considered in periods of heavy training load or recovery from illness or injury.

In summary, the B-complex vitamins are indispensable cofactors in energy metabolism, RBC synthesis, and neurological function. Deficiencies can impair endurance and recovery, especially in high-demand athletes or those with restricted diets. However, supplementation beyond sufficiency does not consistently yield ergogenic benefits. Thus, there is moderate certainty that B-vitamin sufficiency supports energy metabolism and recovery, but low certainty that supplementation improves performance in already well-nourished athletes. Monitoring risk groups such as female athletes and vegetarians/vegans remains essential.

Vitamin E supplementation can reduce oxidative stress and inflammatory markers, supporting short-term recovery. However, high-dose interventions may impair long-term training adaptations such as mitochondrial biogenesis. Overall, there is low to moderate certainty for antioxidant and recovery benefits, but low certainty for sustained performance enhancement. Dietary sources remain the preferred approach, and chronic high-dose supplementation should be avoided.

Vitamin A influences immune regulation, metabolic control, and mitochondrial biogenesis, with mechanistic data suggesting a potential role in exercise recovery. However, athlete-specific trials are minimal, and findings are inconsistent. The certainty of evidence is low for health-related benefits and very low for direct performance outcomes. Dietary adequacy should be ensured, but supplementation cannot be recommended for ergogenic purposes at present.

Vitamin D deficiency is common among athletes, particularly those involved in indoor sports or during winter seasons. Supplementation reliably improves bone mineral density, reduces the risk of stress fractures, and may enhance musculoskeletal recovery. Effects on performance (strength, aerobic capacity, anaerobic power) remain inconsistent. There is moderate certainty for benefits on bone and injury endpoints in deficient athletes, and low certainty for direct performance gains. Screening and targeted supplementation are recommended for individuals with deficiencies.

Vitamin K supports bone mineralization and vascular function, and may modulate inflammation and iron metabolism. Athlete-specific evidence remains sparse, with limited trials and emerging observational studies. There is low certainty regarding bone and vascular benefits, and very low certainty regarding performance outcomes. Adequate dietary intake should be prioritized, with supplementation reserved for research or clinical contexts.

In addition to vitamin-specific evidence, it is essential to acknowledge that specific athlete subgroups are at a heightened risk for deficiencies. Female endurance athletes require closer monitoring of B-complex vitamins (folate and B12) and vitamin K due to menstrual losses and concerns about bone health. Indoor sports athletes are particularly prone to vitamin D insufficiency during winter or in northern latitudes. Weight-class and combat sport athletes may face antioxidant depletion (vitamins C and E) during rapid weight loss phases, while vegetarian and vegan athletes remain vulnerable to B12 and vitamin D deficiencies. These population-specific considerations underscore the importance of tailored screening triggers—such as stress fractures, recurrent infections, or unexplained fatigue—to inform targeted testing and supplementation strategies.

Female Endurance Athletes:

Risk profile: Higher prevalence of iron, folate, and B12 deficiencies due to menstrual losses and dietary restriction during training. Vitamin D deficiency is also frequent in northern latitudes.

Screening triggers Include Fatigue disproportionate to training load, recurrent illness, stress fractures, and low energy availability.

Practical note: Annual screening for vitamin D, iron/ferritin, folate, and B12; closer monitoring during periods of heavy training or dieting.

Indoor Sports Athletes (e.g., gymnasts, swimmers, basketball players):

Risk profile: High prevalence of vitamin D insufficiency due to limited sun exposure.

Screening triggers: Winter season, frequent indoor training, history of stress fractures or bone pain.

Practical note: Baseline and winter vitamin D status should be assessed; supplementation targeted only if serum 25(OH)D < 75 nmol/L.

Weight-Class and Combat Sports Athletes:

Risk profile: B-complex vitamin insufficiencies (B1, B2, B6) and vitamin C depletion during rapid weight loss or restrictive diets. Increased oxidative stress and immune suppression during weight cutting.

Screening triggers: Recurrent upper respiratory infections, prolonged recovery, and unexplained fatigue during training camps.

Practical note: Nutritional screening is recommended at the start of each competitive season and should be monitored closely during dieting/weight-cutting phases.

Vegetarian and Vegan Athletes:

Risk profile: At risk for vitamin B12 and D deficiency, and potentially low intakes of iron, zinc, and omega-3s. Folate is typically sufficient or high, but may mask B12 deficiency.

Screening triggers: Include neurological symptoms (such as tingling or impaired reaction speed), fatigue, poor recovery, low hemoglobin levels, and suboptimal endurance.

Practical note: Annual screening for B12 and vitamin D; consider methylcobalamin supplementation if levels are borderline or deficient.

## Figures and Tables

**Table 1:** Summary of study designs and evidence base per vitamin (2010–2024).

Vitamin	RCTs	Cohort/Longitudinal	Cross-Sectional	Case–Control	Mechanistic/Experimental	Evidence Notes
Vitamin C	>30	~20	~25	~5	~15	Robust RCT base on oxidative stress, recovery, and cortisol regulation; case–control links to tendon injury and recovery.
B-Complex (B1–B12)	20–30	~15	>30	~4	~20	The evidence is broad but heterogeneous, with case–control data primarily focusing on deficiency versus performance outcomes.
Vitamin E	~20	~10	~18	~3	~12	Short-term antioxidant benefits; case–control analyses suggest impaired adaptation in individuals using high doses.
Vitamin A	<10	~6	~15	~2	~10	Sparse RCTs; case–control studies link deficiency with infection risk and training absence.
Vitamin D	>40	>25	>35	~6	~15	Strongest evidence base; case–control studies show deficiency strongly associated with bone stress injuries.
Vitamin K	<10	~5	~12	~2	~8	Emerging field; case–control work limited to bone density and fracture risk.

**Table 2:** Benefits and risks of vitamin C supplementation. (The arrows indicate elicited physiological response).

Benefits	Risks
↓ Muscle soreness [62]	↓ Mitochondrial adaptations in the case of overdosing [63]
↓ Lipid peroxidation (oxidative stress) [64]	Gastrointestinal distress (nausea, cramps) [65]
↓ Cortisol levels (stress hormone) [66]	↑ Risk of kidney stones (especially oxalate) [67]
↑ Recovery speed [68]	↓ Copper and selenium absorption [69]
↑ Immune response [70]	Hemolysis in G6PD-deficient individuals [70]
↓ Inflammatory markers [70]	↑ Iron absorption (problematic in hemochromatosis) [70]
↑ Collagen synthesis and tendon repair [70]	Prooxidant activity with iron/copper [71]
↓ Risk of illness during heavy training [72]	↓ Endogenous antioxidant enzyme signaling [73]

**Table 3:** The relation between B vitamins, sports activity, and the risk of deficiency.

Vitamin	Name	Major Functions in the Sports Context	Deficiency Risk in Athletes
B1	Thiamine	Carbohydrate metabolism, energy production, fatigue reduction	Combat athletes, high-intensity trainers [125]
B2	Riboflavin	Aerobic metabolism, muscle pain reduction, and recovery	Dieting female athletes [116]
B3	Niacin	NAD/NADP coenzyme production, energy metabolism, and muscle recovery	Endurance and stressed athletes [128]
B5	Pantothenic Acid	Coenzyme A precursor, fatty acid metabolism	Limited evidence, needs more research [129]
B6	Pyridoxine	Immune support, muscular endurance, energy metabolism	Athletes with high metabolic turnover [130]
B7	Biotin	Carboxylation in metabolism (fatty acids, carbs)	Unknown in athletes [131]
B9	Folate	Amino acid metabolism, homocysteine control, cardiovascular health, and inflammation reduction	Vegans/vegetarians, female elite athletes [132]
B12	Cobalamin	Red blood cell production, DNA synthesis, oxygen transport, and cognitive function	Vegans/vegetarians, endurance athletes [125]

**Table 4:** Effective dosing of B vitamins.

Vitamin	Athlete Evidence and Performance Findings	Typical Studied Dose in Athletes	Duration Studied	Performance Domain Reported	Biomarkers Reported	Sport Applications
B1 (thiamine)	Mechanistic role in carbohydrate decarboxylation; limited direct athlete RCT evidence suggesting benefit when included in multivitamin/B-complex formulations that reduced fatigue symptoms in athletes and active adults [88,111,133]	20 mg/day in a professional athlete B-complex trial [133]	28–90 days studied in complex formulations [88,133]	Endurance/fatigue reduction in complex formulations; single-vitamin athlete RCTs lacking [111]	Increased blood bioavailable B1 after complex supplementation in athletes [133]	Useful when carbohydrate metabolism or deficiency is suspected
B2 (riboflavin)	Essential for FAD/FMN cofactors; narrative reviews report little consistent positive effect from isolated riboflavin supplementation in exercise trials [111]	15 mg/day in the athlete B-complex trial [133]	As above [133]	No consistent athlete performance gains reported [111]	Increased serum bioavailable B2 after complex supplementation [133]	Status monitoring recommended; isolated supplementation rarely studied
B3 (niacin)	Central to NAD/NADP metabolism; high pharmacological niacin alters metabolism, but the athlete’s benefit is unclear and evidence is inconsistent [111,134]	30 mg/day in one B-complex athlete protocol [133]	As above [133]	No consistent improvements in measured performance from athlete data [111]	No athlete-specific biomarker improvements attributable solely to niacin were reported in the corpus	Caution—pharmacologic niacin doses have metabolic effects in nonathlete literature [111]
B5 (pantothenic acid)	Required for CoA synthesis; no athlete RCT evidence for single-vitamin ergogenic effect in corpus [111]	10 mg/day in the complex trial [133]	-	Insufficient evidence	-	No athlete-specific guidance available
B6 (pyridoxine/P5P)	Involved in amino acid metabolism; included in B-complex trials that reduced perceived fatigue and metabolic markers when given with other B vitamins [88,133]	15 mg pyridoxal-5-phosphate/day in athlete complex [133]	28–90 days in complex trials [88,133]	Fatigue reduction, potentially recovery when combined with other B vitamins [88],	Increased serum B6 after complex supplementation [133]	Consider where deficiency or heavy protein turnover exists
B7 (biotin)	Mechanistic role in carboxylases; no athlete-specific RCT evidence for performance effects in corpus [111]	1000 µg/day in athlete complex [133]	-	Insufficient evidence	-	Insufficient athlete data
B9 (folate)	Important for haematological adaptation and cell synthesis; folate is included in multimicronutrient interventions for athletes and general populations; specific performance gains unclear unless deficiency present [28,135]	400 µg 5-MTHF/day in athlete complex [133]	-	No direct performance endpoints improved in athletes when not deficient [28,135]	Increased folate biomarkers after supplementation in older/multimicronutrient trials (nonathlete) [136]	Particularly relevant for female athletes and haematological adaptation [28]
B12 (cobalamin)	Required for erythropoiesis and one-carbon metabolism; included in athlete B-complex trials that improved fatigue symptoms when baseline insufficiency was likely [88,133]	1000 µg methylcobalamin/day in athlete complex [133]	-	Fatigue and subjective recovery improved in B-complex trials [133]	Holotranscobalamin and serum B12 increase in multimicronutrient studies (non-athlete) [136]	Consider if vegetarian/low-animal-product athletes or suspected deficiency

Notes: Where the table lists direct athlete trial doses and outcomes, those data derive from a professional athlete pre-post B-complex study and a randomized trial of a B-complex product in healthy active adults [88,133], and from narrative reviews on athlete evidence and mechanisms [111]. For most individual B vitamins, athlete-specific randomized trial evidence of single-vitamin ergogenic effects is lacking [111]. Fat-soluble vitamins.

**Table 6:** Relationships between function, mechanisms, and impact on athletic performance relating to supplementation with vitamin A. (The arrows indicate elicited physiological response).

Function	Mechanism	Impact on Athletes
Lipid metabolism [210,214,215]	↑ CPT1, ↓ SREBP-1c	↑ Fat utilization
Glucose metabolism [216,217,218]	↑ GLUT4, ↓ PEPCK, ↓ G6PC	↑ Insulin sensitivity, ↓ blood glucose
Mitochondrial function [219,220]	↑ PGC-1α, NRF1, UCP2/3	↑ Energy efficiency, ↓ fatigue
Antioxidant defense [221,222,223]	synergy with vitamins: C/E, ↓ ROS	↓ Oxidative stress, ↑ recovery
Immune modulation [224,225,226]	↑ T-cell response, ↑ barrier function	↓ Infections, ↑ training consistency
Muscle repair & recovery [185,227]	↑ Protein synthesis, ↓ inflammation	↑ Recovery, ↑ performance

**Table 7:** Key findings on cross-correlations between vitamin D and sports application.

Aspect	Key Findings	References
Prevalence of deficiency	40–70% of athletes, higher in indoor sports and winter; 30–50% even in outdoor sports	[242,243,244,247]
Functions	Muscle function, bone metabolism, calcium absorption, and immune modulation	[155,234,235]
Effects on performance	Mixed results; possible benefits for aerobic capacity, anaerobic power; limited effect on muscle strength unless deficient	[74,236,248,260]
Effects on musculoskeletal health	Improves bone mineral density, reduces risk of stress fractures, aids recovery from injury	[132,246,252,257]
Recovery	May reduce recovery time post-injury and exercise-induced muscle damage	[249,250,255]
Supplementation	Recommended in deficiency; optimal range 75–125 nmol/L; safe at recommended doses; consider calcium and magnesium co-intake	[25,246,251,265]
Limitations of evidence	Mixed performance outcomes; baseline vitamin D status critical for effect	[236,253]

## Data Availability

No new data were created or analyzed in this study. Data sharing is not applicable to this article.
